# Supranutritional Maternal Organic Selenium Supplementation during Different Trimesters of Pregnancy Affects the Muscle Gene Transcriptome of Newborn Beef Calves in a Time-Dependent Manner

**DOI:** 10.3390/genes12121884

**Published:** 2021-11-25

**Authors:** Wellison J. S. Diniz, Gerd Bobe, Joseph J. Klopfenstein, Yunus Gultekin, T. Zane Davis, Alison K. Ward, Jean A. Hall

**Affiliations:** 1 Department of Animal Sciences, Auburn University, Auburn, AL 36849, USA; 2 Department of Animal and Rangeland Sciences, College of Agricultural Sciences, Oregon State University, Corvallis, OR 97331, USA; gerd.bobe@oregonstate.edu (G.B.); yunus.gultekin@oregonstate.edu (Y.G.); 3 Linus Pauling Institute, Oregon State University, Corvallis, OR 97331, USA; 4 Department of Clinical Sciences, Carlson College of Veterinary Medicine, Oregon State University, Corvallis, OR 97331, USA; joe.klopfenstein@oregonstate.edu; 5 USDA-ARS-Poisonous Plant Research Lab, Logan, UT 84341, USA; zane.davis@ars.usda.gov; 6 Department of Animal Sciences, North Dakota State University, Fargo, ND 58105, USA; alison.ward@ndsu.edu; 7 Department of Biomedical Sciences, Carlson College of Veterinary Medicine, Oregon State University, Corvallis, OR 97331, USA

**Keywords:** beef cattle, fetal programming, maternal nutrition, muscle growth, organic selenium

## Abstract

Selenium (Se) is an essential micronutrient for growth and immune function in beef cattle. We previously showed that supranutritional maternal organic Se supplementation during late pregnancy improves immune function in their newborn calves; however, the effects of maternal organic Se-supplementation on fetal programming during different pregnancy stages have yet to be elucidated. Herein, we investigated the effects of supranutritional maternal organic Se-supplementation in different pregnancy trimesters on their beef calf’s genome-wide transcriptome profiles. Within 12 to 48 h of birth, whole blood and *Longissimus dorsi* (LD) muscle biopsies were collected from calves born to 40 crossbred Angus cows that received, except for the control group (CTR), Se-yeast boluses (105 mg of Se/wk) during the first (TR1), second (TR2), or third (TR3) trimester of gestation. Whole-blood Se concentrations of newborn calves increased from CTR, TR1, TR2 to TR3, whereas muscle Se concentrations of newborn calves were only increased in TR3 group. We identified 3048 unique differentially expressed genes (DEGs) across all group comparisons (FDR ≤ 0.05 and |log2FC| ≥ 1.5). Furthermore, we predicted 237 unique transcription factors that putatively regulate the DEGs. Independent of supplementation trimester, supranutritional maternal organic Se supplementation downregulated genes involved in adaptive immunity in all trimesters. Dependent on supplementation trimester, genes involved in muscle development were upregulated by TR3 Se supplementation and downregulated by TR1 Se-supplementation, and genes involved in collagen formation were downregulated by TR2 Se-supplementation. Supranutritional maternal organic Se supplementation in the last trimester of pregnancy resulted in upregulation of myosin and actin filament associated genes, potentially allowing for optimal muscle function and contraction. Our findings suggest a beneficial effect of supranutritional maternal organic Se supplementation during late gestation on Se-status and muscle development and function of newborn calves.

## 1. Introduction

Selenium (Se) is an essential micronutrient that exerts its functions by incorporating selenocysteine into various selenoproteins, which have oxidoreductase activity [1]. Metabolic functions of selenoproteins include regulation of redox homeostasis, energy homeostasis, and thyroid hormone activation [2]. Selenoproteins can also modulate metabolic functions through transcriptome [3] and epigenome [4] regulation. Because soils in large parts of the US are Se deficient, cattle in these regions are typically Se-supplemented [5]. It is well documented that Se deficiency results in neuromuscular diseases, such as nutritional myodegeneration (white muscle disease) and nutritional muscular dystrophy [6]. Our knowledge about how maternal Se supplementation affects skeletal muscle development is primarily limited to avian and monogastric species [1,7,8].

Current epidemiological studies strongly support an intricate interplay between maternal nutrition and fetal development and programming (reviewed in [9]). The developing fetus is dependent on maternal nutrient availability to meet its nutritional requirements [10]. Thus, an impaired intrauterine environment may alter fetal physiology and metabolic programming with long-term detrimental effects on health and performance [11]. Because of the pivotal involvement of selenoproteins in essential biological functions, an increasing number of studies have focused on their roles during pre- and post-natal maternal nutrition [1,2,7,12,13]. Therefore, we propose that an adequate supply of Se may be important for optimal muscle development of the fetus.

Evidence suggests that biological processes regulating normal growth, development, and nutrient utilization are programmed in utero even during the earliest developmental stages [11,14]. Although the fetal demand for energy and nutrients is greatest during the last trimester [15], maternal nutritional imbalances in early to mid-gestation may negatively impact embryo organogenesis [16]. Primary myogenesis in cattle begins in the embryonic phase, whereas most skeletal muscle fibers are formed during the second trimester, followed by muscle hypertrophy during the last third of gestation [16]. Thus, limited nutrient availability during pregnancy leads to adverse effects on muscle size and intramuscular fat deposition [14,16]. Maternal supranutritional Se supplementation can improve growth of their offspring. In broilers, Gao et al. [1] reported that maternal Se-supplementation increased muscle development by activating myogenic factor. In lambs, maternal supranutritional Se-supplementation improved offspring growth and response to a glucose tolerance test [17]. In Jersey dairy calves, maternal supranutritional Se supplementation improved birth weights and IgG absorption and serum-IgG concentrations [5,18].

Despite increasing interest in the role of maternal Se supplementation on fetal programming and long-term offspring performance in livestock, most of the studies have focused on mid to late gestation Se supplementation [2,5,18,19,20]. Likewise, the main focus of fetal programming has been on macronutrients such as protein or global energy supplementation and (or) restriction [21,22,23]. Furthermore, the mechanisms that underlie the crosstalk between maternal nutrition, including Se supplementation, and offspring developmental programming, are still to be elucidated. Given these limitations, the objective of the current study was to investigate the effects of maternal supranutritional organic Se supplementation in different trimesters of pregnancy on whole blood and muscle Se concentration and muscle gene expression profile of newborn calves. We hypothesized that supranutritional maternal organic Se supplementation increases whole blood and muscle Se concentrations in newborn calves and that supranutritional maternal organic Se supplementation increases expression of genes involved in muscle development; these increases are greater in calves of dams supplemented in the last trimester of pregnancy versus earlier in pregnancy.

## 2. Materials and Methods

### 2.1. Ethics Statement

All experimental procedures were approved by the Oregon State University Institutional Animal Care and Use Committee (ACUP number 2019-0056). All methods were performed following the relevant guidelines and regulations.

### 2.2. Animals and Tissue Collection

This was a prospective clinical trial of 2-years duration (May 2019–June 2021) involving beef calves born to cows that had been supplemented with Se-yeast during various trimesters of pregnancy. Cows were assigned to one of four groups at conception (control and groups 1, 2, and 3 corresponding to trimester of Se-treatment: CTR, TR1, TR2, and TR3, respectively), using a randomized complete block design. These were Angus and Angus-cross cows that had calved at least once previously (*n* = 15 to 25 per group). Cows were bred to one sire using artificial insemination with sexed semen. Cows that did not become pregnant with the sexed semen were then bred using several bulls. Ear tags were used to identify cows.

The cows were located at the Oregon State University Soap Creek ranch, Corvallis, OR. Forage Se concentrations in this region range from 0.06 to 0.11 mg/kg (DM basis) [24,25]. Routine farm management practices [e.g., feeding (pasture and hay), vaccinations, and deworming] were the same for all treatment groups. Except in the CTR group, cows received Se-supplementation during their corresponding pregnancy trimester in the form of three Se-yeast (Phibro Selenium Yeast 2000, Prince Agri Products, Inc., Quincy, IL, USA) boluses per week for 13 weeks, equaling 105 mg Se/wk throughout their treatment trimester. This is five times the upper limit that the National Research Council (NRC) recommends for Se supplementation, but well below the documented toxic threshold (5 mg/kg is defined as the maximum tolerable level in ruminants) [26]. Thus, cows received Se-yeast during the first (TR1), second (TR2), or third (TR3) trimester of gestation. In addition, cows had access to a mineral supplement containing 120 mg/kg Se (US FDA regulations) from Na selenite. Within each group of cows, ten were chosen for repeated measures based on uniform genetic background (black color), medium size, and middle aged (5–9 years). The resulting number of calves that were blood sampled and biopsied per treatment group were as follows: CTR group *n* = 10 (3 females, 7 males), TR1 *n* = 9 (2 females, 7 males), TR2 *n* = 11 (3 females, 8 males), TR3 *n* = 10 (6 females, 4 males).

### 2.3. Whole-Blood and Muscle Collection and Selenium Analysis

Within 12 to 48 h of birth, whole blood (WB) samples were collected from the jugular vein of calves into evacuated EDTA tubes (2 mL; final EDTA concentration 2 g/L; Becton Dickinson, Franklin Lakes, NJ, USA). They were stored on ice and frozen at −20 °C until Se concentrations were measured.

Muscle samples were collected at the same time from the *Longissimus dorsi* (LD) using a Bergstrom biopsy needle (inner diameter: 5 mm). The skin over the biopsy site was shaved, surgically prepped, and 5 mL of 2% lidocaine HCl with epinephrine was injected subcutaneously around the biopsy site to provide a local anesthetic. A 1-cm incision was made through the skin, and the Bergstrom muscle biopsy needle was inserted and used to collect sufficient tissue for analysis (~150 mg). The skin incision was closed with surgical staples, and a topical wound spray was applied to the surgical site. A single dose of flunixin meglumine (2.2 mg/kg body weight) was administered IV immediately after the biopsy was collected to provide analgesia. Muscle biopsies were placed into sterile vials, stored on ice and frozen at −80 °C until Se concentrations and total RNA isolation were performed.

Whole blood and muscle Se concentrations were determined by a commercial laboratory (Utah Veterinary Diagnostic Laboratory, Logan, UT, USA) using an ICP-MS (ELAN 6000, Perkin Elmer, Shelton, CT, USA) method as previously described [27]. Statistical analysis of Se concentrations was performed using PROC GLM in SAS version 9.2 (SAS Institute, Inc., Cary, NC, USA). Fixed effects were the treatment group of the dam and sex of the calf. All tests were two-sided. Statistical significance was declared at *p* ≤ 0.05. Graphs were generated in Prism (version 6.0, GraphPad Software, San Diego, CA, USA).

### 2.4. Total RNA Isolation, Library Preparation, Sequencing, and Data Processing

Total RNA was isolated from the LD muscle using the RNeasy Fibrous Tissue Mini Kit (Qiagen^®^, Germantown, MA, USA), following the manufacturer’s protocol. RNA quality control (quantity and purity) was evaluated using a Nanodrop^®^ spectrophotometer, Agilent 2100 Bioanalyzer^®^, and agarose gel electrophoresis. Strand-specific libraries (poly-A enrichment) were prepared using the NEBNext^®^ Ultra™ II Directional RNA Library Prep Kit for Illumina (New England BioLabs^®^, Ipswich, MA, USA). Paired-end sequencing (150-bp reads) at a depth of 20 M reads/sample was carried out on the Illumina^®^ NovaSeq 600 platform. RNA isolation, library preparation, and RNA sequencing were performed by Novogene Co. (Nanjing, China).

Read quality control (QC) was based on removing sequencing adaptors, low complexity reads, and reads containing low-quality bases. Additionally, reads with a PhredScore lower than 30 were filtered out. Read statistics were estimated and visualized with FastQC v0.11.8 (https://bit.ly/3pCUvar, accessed on 11 January 2021) [28] and MultiQC v1.9 (https://multiqc.info/, accessed on 11 January 2021) [29] software. After QC, clean reads (38 samples) were mapped to the *Bos taurus* reference genome (ARS-UCD 1.2) [30] using the STAR aligner v. 2.7.3a (https://rb.gy/dlgdva, accessed on 11 January 2021) [31]. The –*quantMode GeneCounts* flag from STAR and the annotation information (release 100, *Ensembl*) were used to determine the number of raw counts per gene in each sample. MultiQC was used for post-mapping quality control. Furthermore, exploratory data analyses were based on Principal Component Analysis (PCA) and clustering using the R-packages [32] NOISeq v.2.31.0 (10.18129/B9.bioc.NOISeq) [33], edgeR v.3.30.3 (10.18129/B9.bioc.edgeR) [34], and factoextra v.1.0.7 (https://bit.ly/3s2fZPA, accessed on 11 January 2021) [35].

### 2.5. Transcriptome Analysis

Based on the library sizes and the experimental design, lowly expressed genes (less than 10 counts in 70% of the samples) were filtered out using the edgeR *filterByExpr* function [34]. The gene expression normalization procedure, using the DESeq2 v.1.28.1 (10.18129/B9.bioc.DESeq2) *VST* function, was applied to the remaining genes (*n* = 12,964). The DESeq2 design model included the dam’s age, offspring sex, and treatment group. Differentially expressed genes (DEGs) were identified between the treatments considering the t-test method proposed by Reverter et al. [36] and implemented in the R-package CeTF v.1.0.7 (10.18129/B9.bioc.CeTF) [37]. The differences between the treatments, i.e., TR1 vs. CTR, TR2 vs. CTR, TR3 vs. CTR, were tested, and DEGs were considered significant at an FDR adjusted *p*-value ≤ 0.05 and |log2 FC| ≥ 1.5. The treatments were used as the reference group, and DEGs were classified as up or downregulated based on the log2 fold change direction. Gene annotation was performed using biomaRt v.2.38.0 (10.18129/B9.bioc.biomaRt) [38] based on the *B. taurus Ensembl* database.

**Figure 1:**
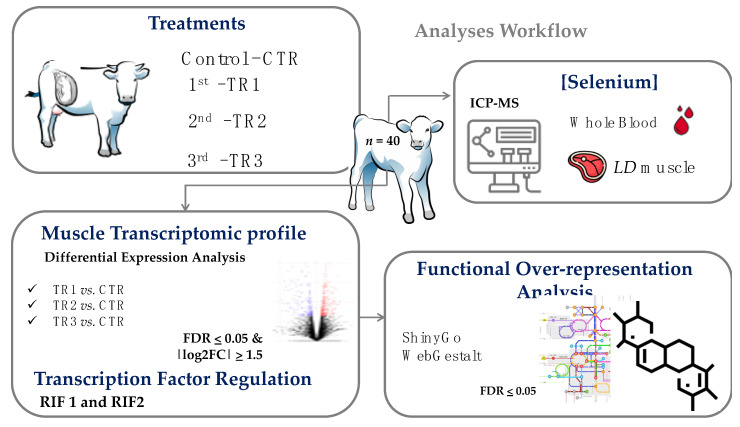
Experimental design and analyses workflow of the study. Pregnant beef cows received, except the control group (CTR), weekly supranutritional selenium-yeast boluses (105 mg Se/wk) during the first (TR1), second (TR2), or third (TR3) trimester of gestation. Whole blood and *Longissimus dorsi* (LD) muscle selenium concentration and LD muscle RNA-Seq were assessed in their newborn calves.

We mined the DEG lists in two different ways: first, we examined the DEG lists of the three Se-supplementation vs. CTR comparisons (Figure 1) and identified shared DEGs among the lists. In a second, more-in-depth analysis, we expanded the number of comparisons from 3 to 14 comparisons. We separately examined DEGs that were upregulated uniquely in one of the three Se-supplementation vs. CTR comparisons, as well as DEGs that were shared in upregulation between two or three Se-supplementation vs. CTR comparisons for a total of 7 comparisons and then did the same for downregulated genes.

To shed light on the biological processes and KEGG pathways that underlie the DEGs, functional over-representation analysis was performed. The queried gene lists included the up or downregulated genes together and separately for each contrast, as well as the overlapping DEGs across treatments. The *B. taurus* annotation was used as background. Over-representation analysis was performed using ShinyGO v0.61 [39] and WebGestalt [40]. Significant results were retrieved after *p*-value adjustment using the Benjamini–Hochberg method (FDR *p* ≤ 0.05).

### 2.6. Analysis of Genes Encoding Transcription Factors with Regulatory Potential

To identify key transcription factors putatively regulating the differentially expressed genes, we used the regulatory impact factor analysis (RIF1 and RIF2) proposed by Reverter et al. [41]. To accomplish this, after mining the AnimalTFDB bovine database v.3.0 [42], we filtered out those genes encoding transcription factors not expressed in our dataset. The remaining genes (*n* = 897) were contrasted separately for each DEG list, comparing treatment vs. CTR [22]. Genes encoding key transcription factors with regulatory potential over DEGs were selected if either one of the two RIF scores was greater than |1.96| of the standard deviation (*p*-value < 0.05) [41]. An overview of the experimental design and the analyses workflow is shown in Figure 1.

## 3. Results

### 3.1. Muscle and Whole-Blood Selenium Concentrations and Body Weights of Newborn Calves

**Figure 2:**
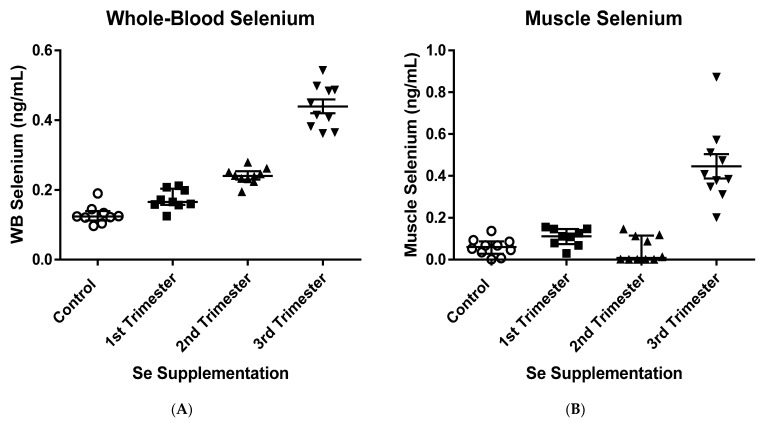
Selenium (Se) concentrations in newborn calves born to dams receiving weekly supranutritional organic Se-yeast boluses (105 mg Se/wk) during different trimesters of pregnancy. (**A**) Whole-Blood Se: All four treatment groups differed at *p* < 0.01 from each other, with the lowest and highest WB-Se concentrations in calves of control (CTR) and in 3rd trimester (TR3) dams, respectively. (**B**) Muscle Se: Calves of Se-supplemented dams in TR3 had higher muscle Se concentrations compared with the two other Se-treatment groups and CTR calves (all *p* < 0.0001), which did not differ from each other (all *p* > 0.10). Horizontal bars refer to mean ± SEM; dots refer to individual calf values.

Whole-blood (WB) and muscle Se concentrations of calves at birth are shown in Figure 2. Supranutritional maternal Se supplementation during gestation was effective at increasing WB-Se concentrations of calves at birth in all three Se-supplemented groups (all *p* < 0.01) compared with the CTR group (0.131 ± 0.012 ng/mL; mean ± SEM). Among the treatments, the highest WB-Se concentrations were observed in calves of Se-supplemented dams in TR3 (0.437 ± 0.012 ng/mL). Medium and low WB-Se concentrations were observed in calves from dams receiving Se supplement in TR2 (0.250 ± 0.012 ng/mL) and TR1 (0.180 ± 0.012 ng/mL), respectively. Muscle Se concentrations were increased only in calves from dams receiving Se supplement in TR3 (0.443 ± 0.033 ng/mL). In these calves, concentrations of muscle Se were similar to WB-Se concentrations (0.437 ± 0.012 ng/mL). The other three groups had similar muscle Se concentrations (CTR: 0.062 ± 0.033 ng/mL; TR1: 0.116 ± 0.036 ng/mL; TR2: 0.059 ± 0.035 ng/mL). Female calves had higher WB-Se concentrations than male calves (female: 0.263 ± 0.010 ng/mL vs. male: 0.236 ± 0.008 ng/mL; *p* = 0.04). Although muscle Se concentrations were changed by a similar magnitude as WB-Se concentrations, differences based on sex were not significant because of the larger SEMs (female: 0.185 ± 0.028 ng/mL vs. male: 0.155 ± 0.022 ng/mL; *p* = 0.40).

Body weights were lower in calves from the TR1 group (35.9 ± 1.1 kg). Calves of the other three groups had similar body weights (CTR: 39.8 ± 0.9 kg; TR2: 40.0 ± 0.9 kg; TR3: 39.1 ± 0.9 kg). Female calves had lower body weights than male calves (female: 36.2 ± 0.7 kg vs. male: 41.2 ± 0.6 kg; *p* < 0.0001).

### 3.2. RNA-Seq Analysis of Muscle Biopsies of Newborn Calves

Sequencing yielded on average 21.4 million reads per sample, of which 97.6% of reads were uniquely mapped to the *B. taurus* genome (Table S1). After QC, 38 samples (nine or ten per treatment) and 12,964 genes were analyzed.

#### 3.2.1. Comparison of the Three Se-Supplemented Groups versus Control

We identified 3048 unique differentially expressed genes (DEGs) with Ensembl IDs across all three TRT vs. CTR comparisons out of 12,946 (24%) tested genes (FDR ≤ 0.05 and |log2FC| ≥ 1.5). The comparison between TR1 vs. CTR revealed 694 up and 677 downregulated genes, respectively (Table S2). The comparison between TR2 vs. CTR showed more genes down than upregulated by Se-supplementation with 636 and 704 up and downregulated genes (Table S3). The comparison between TR3 vs. CTR showed more genes up than downregulated by Se-supplementation with 763 and 656 up and downregulated genes (Table S4).

**Figure 3:**
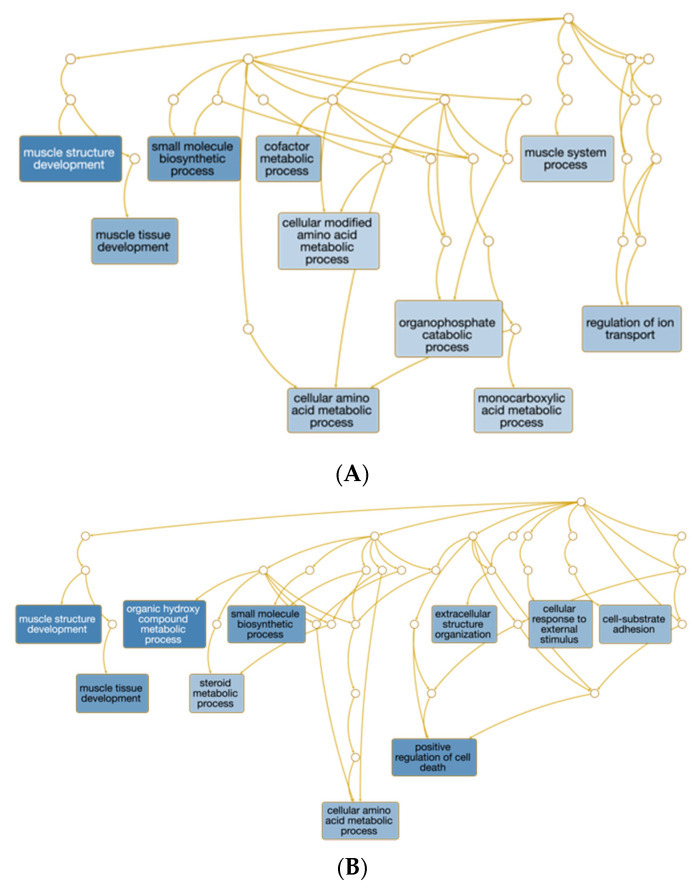

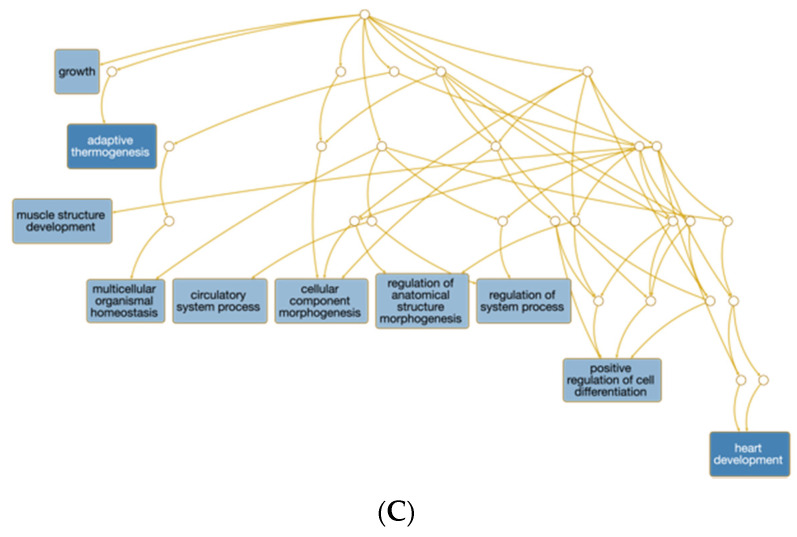
WebGestalt directed acyclic graph of the biological processes over-represented by the differentially expressed protein-coding genes. Differentially expressed genes were identified based on the contrasts between calves from dams in the control group (CTR) with those that received weekly supranutritional Se-yeast boluses (105 mg Se/wk) during the first (TR1), second (TR2), or third (TR3) trimester of gestation. Only the top ten significant over-represented terms containing five or more genes are shown. The darker the blue background color in the squares, the more significant the term is (FDR < 0.05). (**A**) TR1 vs. CTR, (**B**) TR2 vs. CTR, and (**C**) TR3 vs. CTR.

The top ten over-represented GO biological processes (GO terms are italicized) for each of the three TR vs. CTR comparisons are shown in Figure 3 and Table S5 When not considering the direction of change, *muscle structure development* was shared among all three comparisons. The biological processes of *muscle tissue development*, *small molecule biosynthetic process*, and *cellular amino acid metabolic process* were shared between TR1 vs. CTR and TR2 vs. CTR. The top 3 over-represented GO biological processes of TR3 vs. CTR (*growth*, *adaptive thermogenesis*, and *heart development*) were unique to TR3 vs. CTR.

**Figure 4:**
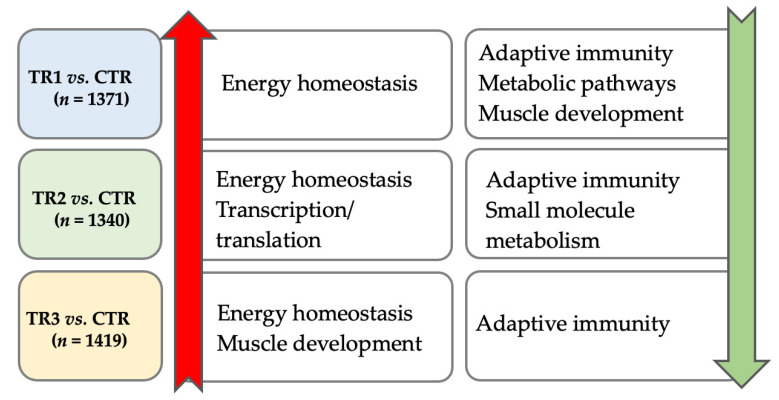
Summary of functional over-representation and gene enrichment analysis of differentially expressed protein-coding genes (DEGs) from the three Se-supplementation vs. CTR comparisons using ShinyGO v0.61 and WebGestalt. Differentially expressed genes were identified based on the contrasts between calves from dams in the control group (CTR) with those that received weekly supranutritional Se-yeast boluses (105 mg Se/wk) during the first (TR1), second (TR2), or third (TR3) trimester of gestation. Numbers under the treatment comparisons represent DEGs for each of the three Se-supplementation vs. CTR comparisons (i.e., TR1 vs. CTR, TR2 vs. CTR, and TR3 vs. CTR). The three comparisons shared biological processes and pathways involved in energy homeostasis being upregulated and biological processes and pathways involved in adaptive immunity being downregulated. Whereas biological processes and pathways involved in muscle development were upregulated in newborn calves from TR3 dams, they were downregulated in newborn calves from TR1 dams.

The impact of Se supplementation separately for up and downregulated genes are shown in Figure 4. In Tables S6 and S7, we show the top 30 GO biological processes that were significantly up or downregulated for each of the comparisons. In comparison to the aforementioned analysis that did not consider direction of change in gene expression, there was no overlap among all three TR vs. CTR comparisons. Four upregulated GO biological processes were shared among two TR vs. CTR comparisons (TR1 and TR2 vs. CTR: *cellular response to chemical stimulus*; *negative regulation of biological process*; *negative regulation of cellular process*; and TR2 and TR3 vs. CTR: *positive regulation of cellular process*), and seven downregulated biological processes were shared among two TR vs. CTR comparisons (TR2 and TR3 vs. CTR: six immune response categories; and TR1 and TR2 vs. CTR: *small molecule metabolic process*). The GO biological processes were exclusively upregulated for TR2 vs. CTR (30 upregulated and 0 downregulated) and TR3 vs. CTR (2 upregulated and 0 downregulated) (FDR ≤ 0.05). For the TR2 vs. CTR comparison, 23 out of the 30 upregulated biological process categories included the terms *regulation* or *positive regulation*, indicating positive regulation of gene expression and RNA biosynthetic processes. For the TR3 vs. CTR comparison, the two upregulated biological process categories were *cellular development process* and *developmental process*, indicating muscle growth promoting effects of TR3. In comparison to TR2 or TR3 vs. CTR, GO biological processes were exclusively downregulated for TR1 vs. CTR (0 upregulated and 12 downregulated). For the TR1 vs. CTR comparison, ten out of the 12 identified downregulated biological process categories included the term *muscle*, suggesting an inhibitory effect of TR1 on skeletal muscle development and muscle cell differentiation. The two remaining biological process categories included the term *small molecule*.

In Tables S8 and S9, we show the top 10 biochemical KEGG pathways that were significantly up or downregulated for each comparison. All three TR vs. CTR comparisons shared upregulation of energy homeostasis signaling pathways (*AMPK signaling pathway* and *insulin resistance*). In addition, TR1 and TR2 vs. CTR comparisons shared upregulation of two additional energy metabolism-related pathways (*PPAR signaling pathway* and *FoxO signaling pathway*) and *ferroptosis*. All three TR vs. CTR comparisons showed downregulation of four immune response-related pathways (*antigen processing and presentation*, *graft-versus-host disease*, *allograft rejection*, and *viral myocarditis*). Furthermore, TR1 and TR2 vs. CTR comparisons shared downregulation of biosynthetic pathways (*glycine*, *serine and threonine metabolism* and *metabolic pathways*). The KEGG pathways were primarily downregulated in all three TR vs. CTR comparisons (9 comparisons were downregulated and 1 comparison was upregulated) (FDR ≤ 0.05). All pathways for TR2 vs. CTR were downregulated and immune associated (*antigen processing and presentation*; *phagosome*); the same was true for TR3 vs. CTR (*antigen processing and presentation*, *graft-versus-host disease*, *allograft rejection*, *Epstein-Barr virus infection*, and *viral myocarditis*). TR1 vs. CTR had the only upregulated pathway (*PPAR signaling pathway*). In addition, TR1 vs. CTR had downregulated immune-associated pathways (*antigen processing and presentation*, *graft-versus-host disease*, and *allograft rejection*), as well as downregulated metabolic pathways (*glycine, serine and threonine metabolism*, *metabolic pathways*, *biosynthesis of amino acids*, and *carbon metabolism*).

#### 3.2.2. Unique and Shared Changes between the Se-Supplementation Groups versus Control

**Figure 5:**
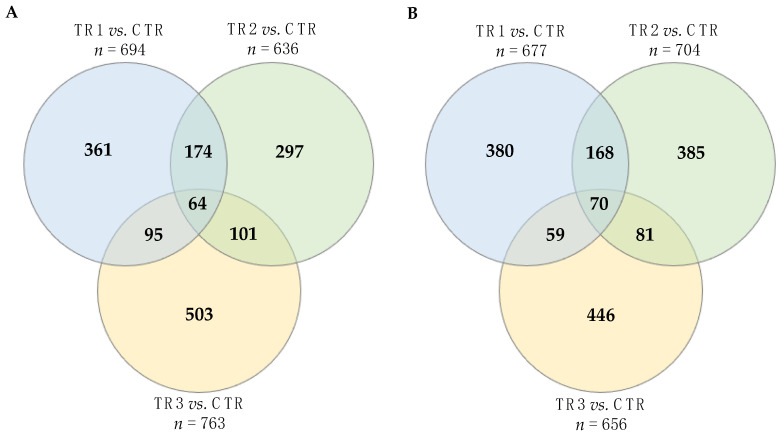
Unique and overlapping differentially expressed genes (DEGs) across treatments: (**A**) upregulated genes and (**B**) downregulated genes. DEGs were identified based on the contrasts between calves from dams in the control group (CTR) with those that received weekly supranutritional Se-yeast boluses (105 mg Se/wk) during the first (TR1), second (TR2), or third (TR3) trimester of gestation. The six numbers outside the circles represent number of (**A**) up or (**B**) downregulated DEGs for each of the three Se-supplementation vs. CTR comparisons (i.e., TR1 vs. CTR, TR2 vs. CTR, and TR3 vs. CTR). The 14 numbers inside the circles represent number of DEGs that were (**A**) up or (**B**) downregulated uniquely in one of the three Se-supplementation vs. CTR comparisons, as well as DEGs that were shared in up or downregulation between two or three Se-supplementation vs. CTR comparisons.

A total of 64 genes were upregulated in all three treatments vs. CTR comparisons and a total 70 genes were downregulated (Figure 5; Table S10). A total of 174 DEGs were upregulated in both TR1 and TR2 vs. CTR but not in TR3 vs. CTR and 168 DEGs were downregulated. In comparison to TR1 and TR2 vs. CTR, less DEGs were uniquely shared between TR2 and TR3 vs. CTR (101 upregulated and 81 downregulated) and between TR1 and TR3 vs. CTR (95 upregulated and 59 downregulated).

A total of 361 DEGs were only upregulated in TR1 vs. CTR and a total of 380 DEGs were only downregulated in TR1 vs. CTR. A total of 297 DEGs were only upregulated in TR2 vs. CTR and a total of 385 DEGs were only downregulated in TR3 vs. CTR. A total of 503 DEGs were only upregulated in TR3 vs. CTR and a total of 446 DEGs were only downregulated in TR3 vs. CTR.

**Figure 6:**
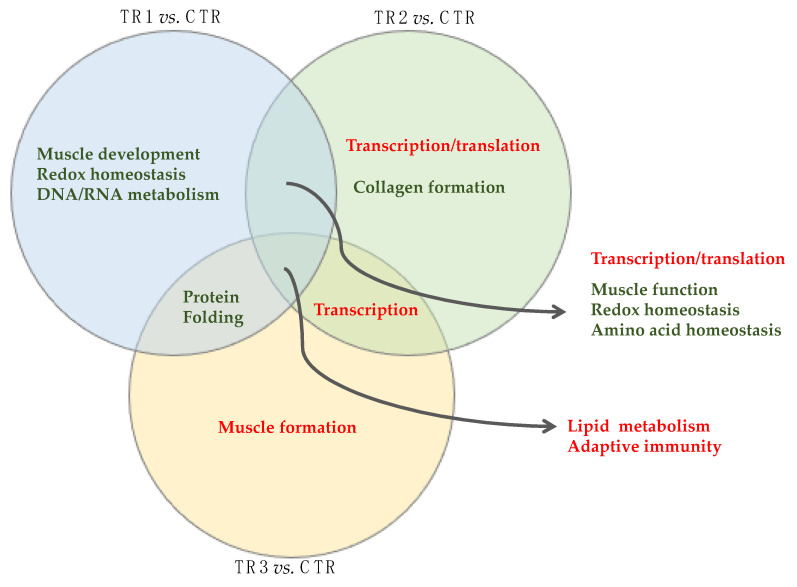
Summary of functional over-representation and gene enrichment analysis of the unique and shared differentially expressed protein-coding genes (DEGs) from the three Se-supplementation vs. CTR comparisons analyzed using ShinyGO v0.61 and WebGestalt. Differentially expressed genes were identified based on the contrasts between calves from dams in the control group (CTR) with those that received weekly supranutritional Se-yeast boluses (105 mg Se/wk) during the first (TR1), second (TR2), or third (TR3) trimester of gestation (i.e., TR1 vs. CTR, TR2 vs. CTR, and TR3 vs. CTR). Next, genes were allocated into seven groups: uniquely changed in direction by TR1, uniquely changed in direction by TR2, uniquely changed in direction by TR3, uniquely changed in same direction by TR1 and TR2, uniquely changed in same direction by TR2 and TR3, uniquely changed in same direction by TR1 and TR3, and uniquely changed in same direction by TR1, TR2, and TR3. Up and downregulated biological processes are color coded as red and green, respectively. All three contrasts shared biological processes and pathways involved in lipid metabolism (upregulated) and biological processes and pathways involved in adaptive immunity (downregulated). Whereas biological processes and pathways involved in muscle development were upregulated in newborn calves from TR3 dams, they were downregulated in newborn calves from TR1 dams. Genes involved in collagen formation were downregulated by TR2 Se-supplementation. In addition, TR1 and TR2 shared downregulation of genes involved in redox and amino acid homeostasis.

Gene Ontology enrichment analysis results are shown in Figure 6. Independent of supplementation trimester, genes involved with lipid metabolism were primarily upregulated (16 upregulated genes: *ABCA6, ARNTL, ARSG, ACACB, ACOT11, ANKRD1, CPT1A, ERFE, F2, GPAT3, LIPE, PCK1, NR4A3, PNPLA2, OSBPL11,* and *SLC22A4;* and 4 downregulated genes: *ARRDC3, C1qTNF3, ZBTB7C,* and *SLC44A3*). Independent of supplementation trimester, pathways involved in the immune response were downregulated with the strongest impact on genes partaking in the antigen processing and presentation process (all 12 downregulated genes: *CD74, BOLA-DMA, BOLA-DMB, BOLA-DQA2, BOLA-DQA5, BOLA-DQB, BOLA-DRA, BOLA-DRB3, BOLA-NC1, COQ2, MRC1,* and *B2M*). At the cellular level, these genes are involved in the MHC protein II complex and the plasma membrane complex (all FDR *p* < 0.001).

TR1 and TR2 vs. CTR shared upregulation of both positive and negative regulatory genes of biological processes and downregulation of genes involved in muscle structure and contraction, genes involved in redox homeostasis, and genes involved in amino acid homeostasis. TR2 and TR3 vs. CTR shared upregulation of positive regulatory genes of biological processes, specifically transcription, and TR1 and TR3 vs. CTR shared downregulation of genes involved in protein folding.

For TR1 vs. CTR alone, there were no KEGG pathways that were upregulated at FDR *p* < 0.01. Muscle development genes were downregulated for TR1 vs. CTR. At the cellular level, genes involved in muscle structure and contraction were downregulated at *FDR p* < 0.01. In addition, we observed downregulation of genes involved in redox homeostasis as well as genes involved the DNA/RNA metabolism. For TR2 vs. CTR, we observed that genes involved in extracellular matrix formation, including collagen, were downregulated. Genes involved in biosynthetic processes were upregulated for TR2 vs. CTR. At the cellular level, these genes were involved in the eukaryotic RNA translation (pre)initiation stages. For TR3 vs. CTR, pathways involved in organ and tissue development, including muscle, were upregulated. At the cellular level, genes were primarily involved in the myosin complex and the cytoskeleton (e.g., actin and cytoskeletal protein binding; all FDR *p* < 0.01). The serine, threonine, and glycine KEGG pathway was also upregulated (FDR *p* = 0.008).

### 3.3. Transcription Factors Putatively Regulating the Differentially Expressed Genes

We focused on transcription factors as potential modulators of differential gene expression. Based on the RIF metrics, 235 (26%) out of the 897 genes encoding transcription factors were identified as potential modulators of differential gene expression. These 235 transcription factors covered 37 transcription factor families. The comparison between TR1 vs. CTR revealed 86 transcription factors, of which 59 were unique to this comparison (Table S11). The comparison between TR2 vs. CTR revealed 90 transcription factors, of which 69 were unique (Table S12), and the comparison between TR3 vs. CTR revealed 92 transcription factors, of which 74 were unique (Table S13). Only *ZNF511* and *ZNF768* were shared as regulators among all comparisons. A total of 14 transcription factors were shared uniquely between TR1 and TR2 vs. CTR, 5 transcription factors were shared between TR2 and TR3 vs. CTR, and 11 transcription factors were shared between TR1 and TR3 vs. CTR.

Thirteen transcription factor coding genes were both DEGs and putative regulators of DEGs (based on RIF metrics) for TR1 vs. CTR; 21 genes were both DEGs and putative regulators of DEGs for TR2 vs. CTR, and eight genes were both DEGs and putative regulators of DEGs for TR3 vs. CTR (Table S14). The over-represented biological processes included regulation of *RNA biosynthetic process*, *regulation of transcription*, *regulation of gene expression*, and *RNA metabolic process* (FDR < 0.05; Figure S1). Six, two, and 29 biological pathways were over-represented for TR1, TR2, and TR3 vs. CTR, respectively (FDR *p* ≤ 0.05). While *nuclear receptor* was a shared pathway among all the comparisons, the *B Cell Receptor Signaling Pathway* was common between TR1 and TR2. Most of the pathways were over-represented in TR3 and included *adipogenesis* (shared with TR1); *IL-4, IL-5, IL-7 and IL-9 signaling pathways*; and *thyroid stimulating hormone* (Figure S2).

## 4. Discussion

A growing number of studies have shed light on the role of macro and micronutrients in fetal metabolic programming through genome regulation [1,21,42]. Among the micronutrients, selenoproteins are involved in regulation of energy metabolism and oxidative homeostasis [2]. However, despite increased attention on the role of Se in maternal nutrition [1,2,5,8], little is known about the interplay of maternal Se-supplementation and the genomic mechanisms that underlie fetal programming. Herein, we investigated the effects of supranutritional maternal Se-yeast supplementation in different trimesters of gestation on the newborns calf’s genome-wide muscle transcriptome. In our study, except for the CTR group, all TR groups received five times the maximal FDA-permitted level of Se. Furthermore, all groups had access to Na selenite mineral supplement *ad libitum* throughout this study; thus, all newborn calves were Se-replete and did not show visual signs of Se-deficiency (i.e., white muscle disease or stiff muscle disease) at birth. The normal range of WB-Se in Se-replete calves is 100–250 ng/mL [43].

In calves, supranutritional maternal organic Se-supplementation during the first trimester decreased birth weights compared with CTR, but had no impact on birthweights when maternal supplementation was in the second or third trimester. We previously reported that supranutritional maternal Se-yeast supplementation improved lamb growth [44] and that Se-supplementation during the backgrounding period increased body weight in weaned beef calves [27]. Furthermore, we reported that maternal Se-yeast supplementation of Jersey dairy cows in the last 8 weeks of gestation improved calf birth weights and 14-day body weights [5]. Maternal organic Se-supplementation of beef cows during the last 8 to 12 weeks of gestation by feeding Se-enriched forages increased WB-Se concentrations in their offspring, but did not affect birthweights [18]. In summary, while the effects of source, level, and time of Se supplementation on animal performance are still debatable [2,5], we conclude that maternal supranutritional supplementation of Se during the latter stages of pregnancy does not impact fetal growth; however, maternal supplementation during the first trimester of pregnancy may decrease birth weights.

To the best of our knowledge, this is the first study investigating the effects of maternal supranutritional Se-yeast supplementation (primarily in the form of selenomethionine) during different trimesters of gestation in beef cows on the LD muscle gene transcriptome of their newborn calves. Herein, our discussion will focus on the role of organic Se in gene expression effects that are trimester of supplementation specific (muscle development and energy metabolism) and trimester of supplementation nonspecific (adaptive immunity and lipid metabolism). Because there is a lack of knowledge in the literature regarding the interplay between organic Se and fetal programming, as well as its potential effects in fetal development in cattle, we will draw a parallel between our findings and the current research in other livestock species and animal models.

### 4.1. Supranutritional Maternal Se-Yeast Supplementation Effects on the Longissimus Dorsi Muscle Transcriptome That Are Dependent on the Trimester of Supplementation

The fetal period is critical for muscle development as unbalanced or inadequate maternal nutrition limits myogenesis [12,16,21,45]. Although primary myogenesis starts during the embryonic stage, most skeletal muscle fibers are formed in mid-gestation, followed by muscle hypertrophy during the last trimester of pregnancy [16].

Findings in the current study revealed that the effect of supranutritional maternal organic Se supplementation may differ by pregnancy trimester. In calves from cows that received organic Se supplementation in the first trimester of pregnancy, genes involved in muscle development (specifically muscle structure related genes) were downregulated, which was linked to lower birth weights. In comparison, supranutritional maternal organic Se supplementation in the last trimester of pregnancy resulted in upregulation of genes involved in muscle function (specifically myosin and actin filament associated genes), potentially allowing for optimal muscle function and contraction. In calves from cows that received organic Se supplementation in the second trimester of pregnancy, genes involved in extracellular matrix development (specifically collagen-related genes) were downregulated. The interplay between Se supplementation and muscle metabolism has been recognized in several studies [1,13], although its precise role remains unclear [6]. It appears that supranutritional maternal Se-yeast supplementation in the last trimester may improve muscle function of newborn calves. Furthermore, supranutritional maternal Se-yeast supplementation in the last trimester may prevent Se-deficiency linked diseases (i.e., rigid/stiff lamb syndrome and white muscle disease). Selenium deficiency limits muscle function and can be treated acutely using Se-injections; however, long-term Se-deficiency results in myodegeneration and replacement of muscle fibers with fibrous connective tissue and calcification [46]. Thus, the amount of Se needed for optimal muscle function may be higher than current recommendations.

Decreased transcription and translation is an obvious link to decreased muscle growth and birth weight in newborn calves. Yim et al. [13] observed lower amino acid and nucleotide levels in Se-deficient mice. Gao et al. [1] reported that maternal Se supplementation led to improved skeletal muscle growth through protein synthesis activation in chicks. Our functional analysis revealed a link between less muscle growth and cellular amino acid (AA) and nucleotide synthesis. The calves from TR1 supplemented cows had downregulation of both amino acid metabolism and DNA/RNA metabolic processes. In comparison, upregulation of glycine, serine and threonine metabolism (7 genes upregulated: *ALAS1, AOC2, DLD, GLYCTK, PHGDH, PSPH,* and *SHMT2*; *FDR p* = 0.008), precursors for the synthesis of proteins and nucleotides, were linked to upregulation of muscle development in TR3 calves. Thus, supranutritional maternal Se-yeast supplementation during the first trimester may impede muscle growth by limiting amino acid and nucleotide synthesis, whereas supranutritional maternal Se-yeast supplementation during the last trimester may promote muscle function by increasing glycine, serine and threonine metabolism.

Myogenic regulatory factors are an important control element for muscle growth [47]. Gao et al. [1] showed that maternal Se supplementation of broiler hens increased expression of myogenic factors (*MYF5, MYOD, MYOG*) in breast muscle of their offspring. Diniz et al. [22] reported that these transcription factors differentially connected and negatively affected myogenesis in nutrient-restricted bovine fetuses. In the current study, the effect of maternal supranutritional Se-yeast supplementation on myogenic gene expression reflected the supplementation trimester-dependent change for muscle development. Whereas muscle development and myogenic regulatory factors (i.e., *MYF3, MYF6, MYH1, MYH3, MYH6, MYOG, MYL3*) were all downregulated in calves from TR1 dams, muscle development and myogenic regulatory factors (i.e., *MYF3, MYH1, MYH3, MYH6, MYOG, MYL3*) were all upregulated in calves from TR3 dams, and some of the myogenic regulatory factors (*MYF6* and *MYH1*) were upregulated in calves from TR2. Thus, myogenic transcription factors may in part explain the differential effects of maternal Se-yeast supplementation on the LD muscle transcriptome.

We identified signaling pathways (i.e., *FOXO signaling pathway* and *AMPK signaling pathway*) in LD muscle of newborn beef calves as potential molecular targets for muscle formation and function via supranutritional maternal Se-yeast supplementation. Among the FOXO isoforms important for skeletal muscle, we identified *FOXO1*, *FOXO3,* and *FOXO4* as upregulated in TR1 and TR2 calves. In comparison, *FOXO1* was downregulated in TR3 and *FOXO6* was upregulated in TR3 calves. In addition, the FOXO-responsive genes *MDM2* and *PCK2* were downregulated in calves from TR1 dams and upregulated in calves from TR3 dams. Decreased muscle mass and fiber atrophy have been associated with the upregulation of *FOXO1* and *FOXO3* [48], whereas *FOXO6* mediates insulin action on target gene expression [49]. In broilers, maternal Se supplementation promoted mTOR and FOXO phosphorylation and activation [1]. Thus, maternal organic Se-yeast supplementation may, in part, impact muscle growth and the LD muscle transcriptome via the *FOXO signaling pathway*. In the current study, *PRKAG3* (i.e., AMPK) was upregulated in calves from TR3 supplemented cows and downregulated in TR1 supplemented cows. In addition, a total of 13 AMPK target genes (*ACACB, CAB39L, CPT1A, CREB1, LIPE, PCK1, PCK2, PFKFB4, PRKAG3, PPP2R1B, SLC2A4, SREBF1,* and *ULK1*) were upregulated in TR3 calves. The FOXO family members interact with AMPK and mTOR in response to nutrient availability and control protein synthesis and energy homeostasis [48,50]. Thus, supranutritional maternal Se-yeast supplementation may promote muscle growth by altering the nutrient-sensing FOXO and AMPK axes.

### 4.2. Supranutritional Maternal Se-Yeast Supplementation Effects on the Longissimus Dorsi Muscle Transcriptome That Are Independent of the Trimester of Supplementation

The most striking changes in LD muscle transcriptome that were independent of the trimester of Se supplementation affected pathways involved with immune response. These were downregulated in the LD muscle transcriptome of newborn calves from Se-supplemented cows. The strongest impact was on genes involved in the MHC class II protein complex and the plasma membrane complex (all 12 downregulated genes: *CD74, BOLA-DMA, BOLA-DMB, BOLA-DQA2, BOLA-DQA5, BOLA-DQB, BOLA-DRA, BOLA-DRB3, BOLA-NC1, COQ2, MRC1,* and *B2M*). Muscle myocytes can facultatively express MHC class II proteins in response to cytokine induction and, thus, participate in the adaptive immune response [51,52]. Immune cell infiltrates and inflammation are part of the pathology of white muscle disease [53]. Thus, supranutritional maternal Se-yeast supplementation may prevent proinflammatory processes in the muscle.

We previously have shown in whole-blood neutrophils of ewes that supranutritional Se-yeast supplementation upregulated genes involved in bacterial recognition (*TLR4, L-Sel*, and *IL-8R*) and decreased *PPM1A*, which is a negative regulator of cell stress response pathways. Many of the ewes were clinically diseased with foot rot [54]. In recently weaned, visually healthy beef calves that were supplemented with Se-enriched alfalfa hay during the backgrounding period, *IL-8R* and *L-Sel* were downregulated and *PPM1A* was not affected by Se-supplementation [55]. In the current study, none of these genes were differentially expressed by supranutritional Se supplementation. We hypothesize that supranutritional maternal Se-yeast supplementation may downregulate LD muscle genes involved in immune responses to provide more energy and nutrients for muscle growth and function; i.e., supranutritional Se may support nutrient sensing for optimal growth.

Whereas pathways involved in antigen processing and presentation were downregulated, pathways involved in lipid metabolism were primarily upregulated (16 upregulated genes: *ABCA6, ARNTL, ARSG, ACACB, ACOT11, ANKRD1, CPT1A, ERFE, F2, GPAT3, LIPE, PCK1, NR4A3, PNPLA2, OSBPL11,* and *SLC22A4;* and 4 downregulated genes: *ARRDC3, C1qTNF3, ZBTB7C,* and *SLC44A3*) in all three Se-supplementation calf groups. The genes serve multiple functions: some are related to fat pad development (*ARRDC*), some are involved in fatty acid oxidation (*ACACB, ACOT11*, *CPT1A, GPAT3,* and *PCK1*), and others in lipid transport and metabolism (*LIPE* and *PNPLA2*). Supranutritional maternal Se supplementation may increase fatty acid oxidation via the AMPK pathway given that AMPK molecular targets (i.e., *ACACB, CPT1A,* and *LIPE*) were upregulated in all supplementations group and that Zhang et al. [56] has reported increased levels of acylcarnitine in pigs fed a high-Se diet. Assessment of a subset of these calves at slaughter indicated a higher number of calves that had superior carcass quality classified as “*Certified Angus Beef^®^*” (CAB) from TR2 and TR3 cows (2 of 6 calves and 3 of 5 calves, respectively) compared with CTR and TR1 cows (0 of 6 calves and 0 of 7 calves, respectively; Fisher’s exact test: *p* = 0.01), indicating potentially better meat quality and marbling with supranutritional maternal Se-yeast supplementation. The CAB certification requires besides consistent sizing, maximum of 1-inch fat thickness, superior muscling and lack of quality deficits, a minimum of 5.8% evenly distributed intramuscular fat content of the LD muscle with medium to fine marbling texture [57]. This indicates potentially better meat quality and marbling with supranutritional maternal Se-yeast supplementation. All but 4 animals were classified as Choice grade; one CTR calf and one TR1 calf were classified as “Select” and one TR1 calf and one TR3 calf were not graded (No roll).

### 4.3. Effects of Supranutritional Maternal Se-Yeast Supplementation on Selenoproteins Gene Expression

We hypothesized that supranutritional maternal Se-yeast may increase Se deposition in LD muscle. Whereas the liver is more responsive to Se supplementation in terms of gene expression [58], the skeletal muscle is the main Se storage site in the form of selenomethionine-containing proteins [59]. In the current study, maternal supranutritional organic Se supplementation increased LD muscle Se concentrations in newborn calves of TR3 dams, indicating the opportunity to have extra Se stored as selenomethionine-containing proteins in TR3 calves.

Whereas Se deficiency dramatically decreases gene expression of selenoproteins, supranutritional Se supplementation of mice had limited effects on selenoprotein expression in liver and brain [13]. In the current study, supranutritional maternal Se supplementation increased muscle gene expression of some selenoproteins. The Se-carrier protein gene *SELENOP* was upregulated in the TR1 vs. CTR and TR3 vs. CTR comparisons. The mitochondrial *SELENOO* gene was upregulated in the TR1 and TR2 groups. Whereas the protein encoded by the *SELENOP* gene is involved in Se homeostasis and transport [60], the role of SELENOO is still under investigation [61]. Nevertheless, it has been suggested that SELENOO protein plays a role in response to oxidative stress and modulates global S-glutathionylation levels in the mitochondria [62]. In addition, the selenoproteins plasma *GPX3* and cytosolic *GPX1* were upregulated in TR1 and TR3 calves, respectively. The glutathione family finely tunes the redox balance to maintain cellular homeostasis [63]. In support, Juszczuk-Kubiak et al. [58] reported that Se-supplemented lambs had increased expression of *GPX1*, *SELENON*, *SELENOW*, and *SELENOP* (12 selenoprotein genes were tested) in LD muscle.

We previously reported that supranutritional organic Se supplementation had a limited effect on selenoprotein gene expression in whole blood neutrophils. Supranutritional organic Se supplementation (via agronomic biofortification of feedstuffs) in weaned beef calves during the backgrounding period did not significantly affect gene expression of five selenoproteins (*SELENOW*, *GPX1*, *GPX4*, *TXNRD1*, and *TXNRD2*) [55]. Supranutritional organic Se-yeast supplementation in ewes increased gene expression of *SEPS1* and *GPX4* and decreased *DIO3* (eight selenoprotein genes were tested) [54]. Gao et al. [1] reported that maternal Se supplementation of chicken increased *SELENOW* gene expression in the breast muscle of their offspring; however, in the current study, *SELENOW* gene expression was not significantly altered by maternal supranutritional Se supplementation in beef cattle nor was it in our previous organic Se supplementation studies.

In summary, maternal supranutritional organic Se supplementation in the last trimester of pregnancy can deposit some of the extra selenomethionine from Se-yeast into the developing muscle of the fetus, but has limited effects on selenoprotein gene expression in LD muscle, except for the Se-transport protein *SELENOP* and some of the more abundant selenoproteins (*GPX1* and *GPX3*). This is to be expected as excess selenomethionine from Se-yeast replaces methionine in skeletal muscle protein synthesis. Muscle selenomethionine is released during proteolysis and can then be converted to selenocysteine for selenoprotein synthesis, if there is a need for increased synthesis of selenoproteins, e.g., because of metabolic stress.

Selenomethionine in proteins may alter the reactivity of protein in which it is incorporated. Replacement of methionine by selenomethionine has limited effects on the structure of proteins but may alter protein reactivity as Se can exchange electrons easier than sulfur [63]. The fact that many of the supranutritional maternal Se-yeast supplementation effects happened in the absence of elevated muscle Se and the strong impact on specific signal transduction pathways (i.e., AMPK signaling pathway and FOXO signaling pathway) suggest that selenomethionine may modulate metabolic functions through methylation, phosphorylation, or both, which will be the focus of future studies.

## 5. Conclusions

Our findings suggest that supranutritional maternal Se supplementation plays a pivotal role in programming muscle gene expression. Additionally, Se differentially modulates offsprings’ muscle gene expression according to the trimester of pregnancy. The results suggest a beneficial effect of Se supplementation during the last third of gestation as the myogenic factors were upregulated. However, further investigation is still needed to confirm this finding and the long-term consequences on offspring muscle development and function.

## Data Availability

All relevant data are within the paper and its Supplementary Information files. All sequencing data is publicly available on NCBI’s Gene Expression Omnibus (GSE189456).

## Supplementary Materials

The following are available online at https://www.mdpi.com/article/10.3390/genes12121884/s1. Table S1: Muscle RNA sequencing summary and mapping statistics. Table S2: Differentially expressed genes between TR1 vs. CTR groups from muscle tissue. Table S3: Differentially expressed genes between TR2 vs. CTR groups from muscle tissue. Table S4: Differentially expressed genes between TR3 vs. CTR groups from muscle tissue. Table S5: Over-represented biological processes of protein coding differentially expressed genes for each of the group contrasts from muscle tissue. Table S6: Over-represented biological processes of upregulated genes for each of the group contrasts from muscle tissue. Table S7: Over-represented biological processes of downregulated genes for each of the group contrasts from muscle tissue. Table S8: Over-represented KEGG pathways of upregulated genes for each of the group contrasts from muscle tissue. Table S9: Over-represented KEGG pathways of downregulated genes for each of the group contrasts from muscle tissue. Table S10: Differentially expressed genes in the same direction shared by all three treatments vs. CTR comparisons. Table S11: Transcription factors identified as putative regulators of differentially expressed genes in muscle for TR1 vs. CTR based on the RIF1 and RIF2. Table S12: Transcription factors identified as putative regulators of differentially expressed genes in muscle for TR2 vs. CTR based on the RIF1 and RIF2. Table S13: Transcription factors identified as putative regulators of differentially expressed genes in muscle for TR3 vs. CTR based on the RIF1 and RIF2. Table S14: Transcription factors identified as differentially expressed and potential key regulators based on the Regulatory Impact Factor metrics. Figure S1: Biological processes over-represented by the key transcription factors. Figure S2: Curated WikiPathways over-represented by the key transcription factors.
