# Supplementary material for: Supranutritional Maternal Organic Selenium Supplementation during Different Trimesters of Pregnancy Affects the Muscle Gene Transcriptome of Newborn Beef Calves in a Time-Dependent Manner
Source: Genes (Basel). 2021 Nov 25;12(12):1884. doi: 10.3390/genes12121884 (PMC8701265; doi:10.3390/genes12121884)
Supplement: Supplementary file 1 [file genes-12-01884-s001.zip › Supplementary Figure_S1.pdf]

Supranutritional Maternal Organic Selenium Supplementation During Different Trimesters of Pregnancy Affects the Muscle Gene Transcriptome of Newborn Beef Calves in a Time-dependent Manner

Wellison J. S. Diniz [1,*], Gerd Bobe [2,3], Joseph J. Klopfenstein [4], Yunus Gultekin [2], T. Zane Davis [5], Alison K. Ward [6] and Jean A. Hall [7,*]

1 Department of Animal Sciences, Auburn University, Auburn, AL 36849, USA
2 Department of Animal and Rangeland Sciences, College of Agricultural Sciences, Oregon State University, Corvallis, OR 97331, USA; gerd.bobe@oregonstate.edu (G.B.); yunus.gultekin@oregonstate.edu (Y.G.)
3 Linus Pauling Institute, Oregon State University, Corvallis, OR 97331, USA
4 Department of Clinical Sciences, Carlson College of Veterinary Medicine, Oregon State University,
Corvallis, OR 97331, USA; joe.klopfenstein@oregonstate.edu
5 USDA-ARS-Poisonous Plant Research Lab, Logan, UT 84341, USA; zane.davis@ARS.USDA.GOV
6 Department of Animal Sciences, North Dakota State University, Fargo, ND 58105, USA; alison.ward@ndsu.edu
7 Department of Biomedical Sciences, Carlson College of Veterinary Medicine, Oregon State University,
Corvallis, OR 97331, USA
* Correspondence: wzd0027@auburn.edu (W.J.S.D.)¿ jean.hall@oregonstate.edu (J.A.H.)

Supplementary Figures

A

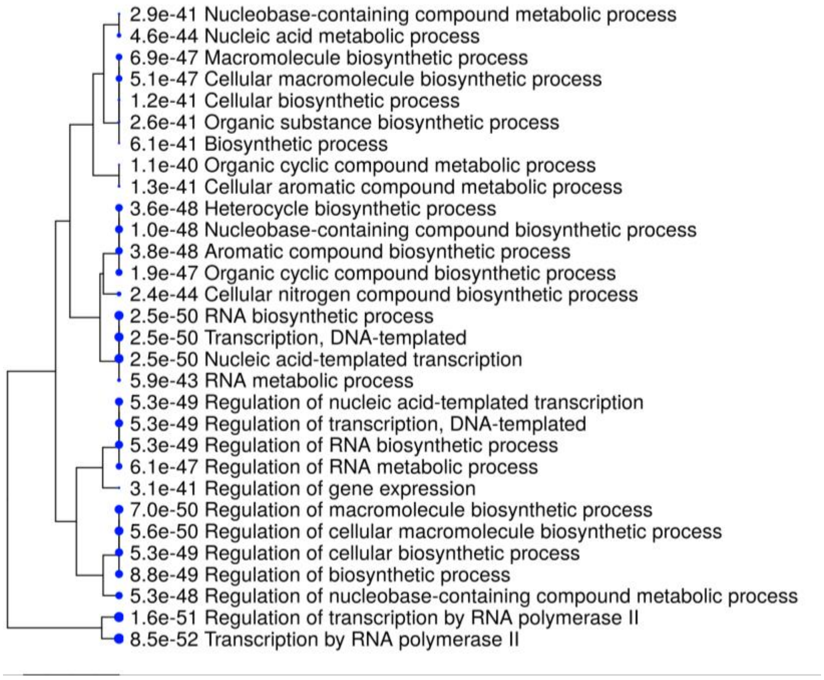

B

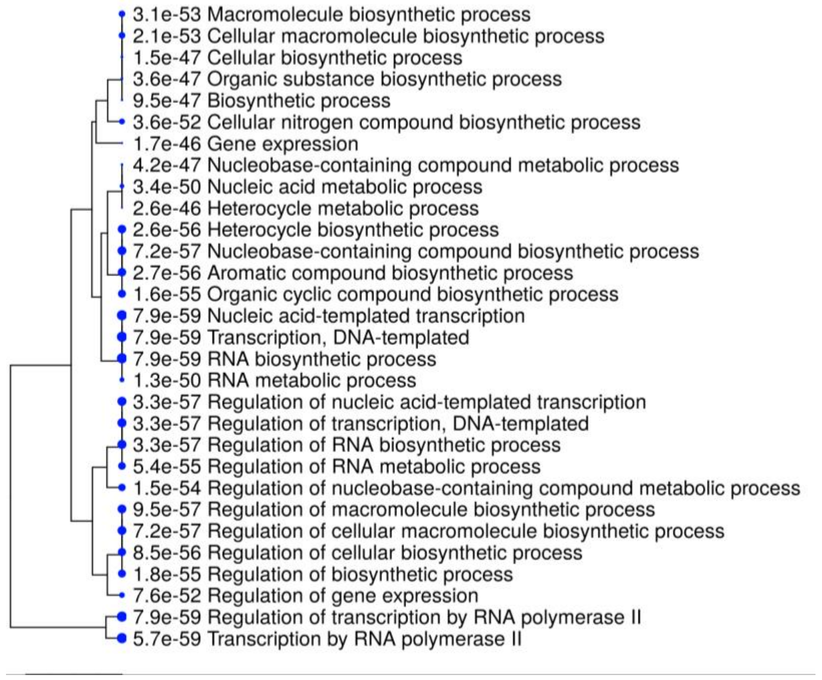

C

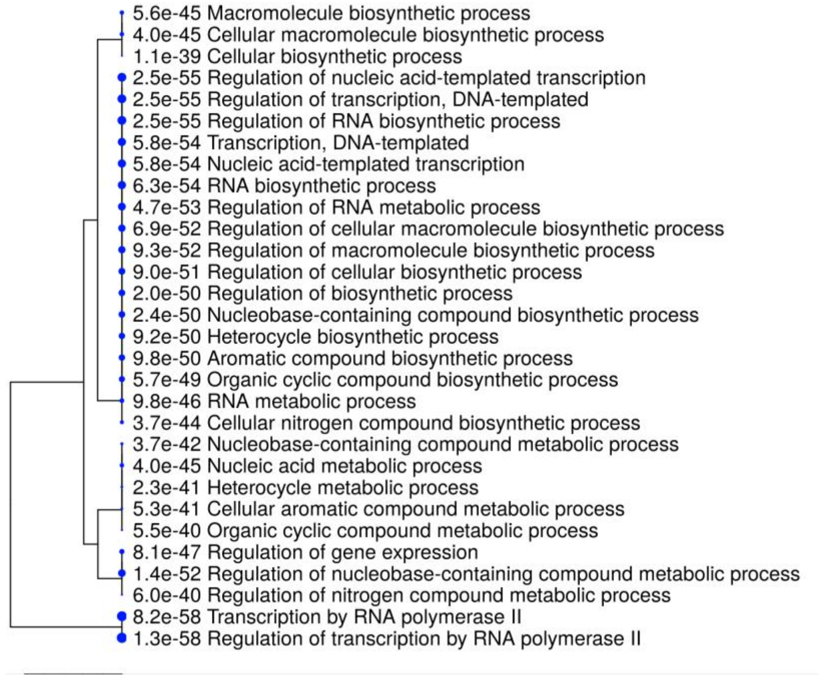

Figure S1. Biological processes over-represented by the key transcription factors. Contrasts were performed separately for each differentially expressed gene, comparing treatment *vs*. control. **A:** First (TR1); **B:** second (TR2); **C:** or third (TR3) trimester of gestation. The bigger the blue dot, the more significant the term is (FDR < 0.05).
